# Cophenetic correlation analysis as a strategy to select phylogenetically informative proteins: an example from the fungal kingdom

**DOI:** 10.1186/1471-2148-7-134

**Published:** 2007-08-09

**Authors:** Eiko E Kuramae, Vincent Robert, Carlos Echavarri-Erasun, Teun Boekhout

**Affiliations:** 1 Yeast Research, CBS-Fungal Biodiversity Centre, Uppsalalaan 8, 3584 CT Utrecht, The Netherlands

## Abstract

**Background:**

The construction of robust and well resolved phylogenetic trees is important for our understanding of many, if not all biological processes, including speciation and origin of higher taxa, genome evolution, metabolic diversification, multicellularity, origin of life styles, pathogenicity and so on. Many older phylogenies were not well supported due to insufficient phylogenetic signal present in the single or few genes used in phylogenetic reconstructions. Importantly, single gene phylogenies were not always found to be congruent. The phylogenetic signal may, therefore, be increased by enlarging the number of genes included in phylogenetic studies. Unfortunately, concatenation of many genes does not take into consideration the evolutionary history of each individual gene. Here, we describe an approach to select informative phylogenetic proteins to be used in the Tree of Life (TOL) and barcoding projects by comparing the cophenetic correlation coefficients (CCC) among individual protein distance matrices of proteins, using the fungi as an example. The method demonstrated that the quality and number of concatenated proteins is important for a reliable estimation of TOL. Approximately 40–45 concatenated proteins seem needed to resolve fungal TOL.

**Results:**

In total 4852 orthologous proteins (KOGs) were assigned among 33 fungal genomes from the Asco- and Basidiomycota and 70 of these represented single copy proteins. The individual protein distance matrices based on 531 concatenated proteins that has been used for phylogeny reconstruction before [14] were compared one with another in order to select those with the highest CCC, which then was used as a reference. This reference distance matrix was compared with those of the 70 single copy proteins selected and their CCC values were calculated. Sixty four KOGs showed a CCC above 0.50 and these were further considered for their phylogenetic potential. Proteins belonging to the cellular processes and signaling KOG category seem more informative than those belonging to the other three categories: information storage and processing; metabolism; and the poorly characterized category. After concatenation of 40 proteins the topology of the phylogenetic tree remained stable, but after concatenation of 60 or more proteins the bootstrap support values of some branches decreased, most likely due to the inclusion of proteins with lowers CCC values. The selection of protein sequences to be used in various TOL projects remains a critical and important process. The method described in this paper will contribute to a more objective selection of phylogenetically informative protein sequences.

**Conclusion:**

This study provides candidate protein sequences to be considered as phylogenetic markers in different branches of fungal TOL. The selection procedure described here will be useful to select informative protein sequences to resolve branches of TOL that contain few or no species with completely sequenced genomes. The robust phylogenetic trees resulting from this method may contribute to our understanding of organismal diversification processes. The method proposed can be extended easily to other branches of TOL.

## Background

Many biological processes can be better understood in the framework of reliable phylogenetic analyses. This is not only true for our understanding of evolutionary systematics and phylogenetics, including TOL, but it will also largely contribute to our understanding of diversification at the subcellular, cellular and organismal levels of integration. One well documented example in this respect is the postulated whole-genome duplication (WGD) that occurred during the evolution of some species belonging to the Saccharomycotina [1]. Only using a correctly inferred phylogenetic TOL it was possible to distinguish between "pre-WGD" and "post-WGD" species of Saccharomycotina. Other examples refer to our understanding of evolution of metabolic pathways [2], structure of genomes [3,4], life styles [5], and pathogenicity [6].

Until recently, our understanding of the (fungal) TOL has been based on two approaches, which basically differ in number of species and genes considered: (1) few genes and large number of species; (2) large number of genes and few species. The clear advantage of the first approach is the availability of many sequences, e.g. of the rDNA locus, in publicly available databases (i.e. National Center for Biotechnology Information – NCBI), and, secondly, it is generally rather easy to generate complete or partial sequences of a few genes for a large number of species. Besides, the rDNA loci have the clear advantage of being universally present in all branches of TOL, universal primers are well known and it has been successfully explored in many branches of TOL. The disadvantage of the rDNA loci, however, is that the deeper branches are usually less supported [7]. As an answer to this, various authors started to include multiple protein coding genes in their phylogenetic analyses [8-10]. Unfortunately, the rationale behind the selection of these protein coding genes is not always clear, and discrepancies and incongruences between individual gene trees may result in unresolved phylogenetic trees [7,8]. This may be due to different evolutionary rates, and/or different origins of the genes, e.g. whether nuclearly encoded (e.g. RPB1 and RPB2) or mitochondrial in origin (e.g. ATP6). In the second approach, large numbers of genes have been used for phylogenetic studies as an attempt to contribute to the first approach described above. This was firstly applied in the prokaryotes [11] and, more recently, in eukaryotes as well [12-14]. A large selection of genes and/or proteins are concatenated and used for inferring phylogenetic relationships, thereby increasing the phylogenetic signal considerably [12,14-17]. However, although this approach resolved the fungal phylogenetic tree [12,14,16,17] it also suffers from some limitations. For instance, it does not take into consideration the evolutionary history of each individual gene and it depends on the availability of complete genome data.

Here, we explored the usefulness of comparing the cophenetic correlation coefficients (CCCs) among distance matrices of individual gene trees in order to make a phylogenetically meaningful selection of orthologs to be considered for further phylogenomics studies as well as large scale TOL and barcoding applications. We used the fungal kingdom as an example as it represents one of the major clades of life with approximately 1.5 million species [18], of which only approximately 80.000 have been described. Moreover, the fungi are morphologically, metabolically and ecologically highly diverse and, importantly, the number of completely sequenced genomes is high among the eukaryotes.

Candidate proteins to be considered for TOL and/or barcoding studies were assessed from 33 fungal proteomes by comparing (i) distance matrices of each individual orthologous protein (KOGs) matrix, (ii) to compare these with that of a well supported guide tree [14], and (iii) analyze for their phylogenetic signal. The method presented here may be universally applied for the selection of markers in various TOL and barcoding studies.

## Results and Discussion

The 33 genomes investigated shared 4852 KOGs from which 70 were single copy proteins. The function of these 70 KOGs was assessed from the *Saccharomyces cerevisiae *genome database [19] (Additional file 1). The corresponding systematic name, standard name, description, chromosome number and knock out phenotype are presented in Table 1 (Additional file 1). Knock out phenotypes of 32 genes were lethal (Table 1) when deleted in *S. cerevisiae *[19], thus suggesting that they code for essential proteins. Genes coding for the 70 KOG proteins are distributed on almost all chromosomes of *S. cerevisiae*, except chromosome VI (Table 1), thus representing the entire genome.

**Table 1:** Correlation values of KOG distance matrices compared to that of KOG2671, KOG functional category, the corresponding single protein KOGs to the systematic name, systematic deletion and chromosome number of ORFs of *Saccharomyce cerevisae *(Sce) [19].

**Correlation value**	**KOG number**	**Sce Systematic name**	**Systematic deletion**	**Chromosome number**
1.00	KOG2671	YOL124c	viable	XV
0.93	KOG0340	YHR169w	inviable	VIII
0.91	KOG4089	YDR405w	viable	IV
0.91	KOG0173	YOR157C	inviable	XV
0.91	KOG2728	YIL083c	inviable	IX
0.90	KOG3111	YJL121c	viable	X
0.89	KOG3800	YDR460w	inviable	IV
0.89	KOG3024	YOR164c	viable	XV
0.89	KOG0816	YKL009w	viable	XI
0.89	KOG2905	YGR005c	inviable	VII
0.89	KOG3013	YHR069c	inviable	VIII
0.89	KOG1416	YNL062c	inviable	XIV
0.88	KOG2299	YNL072w	viable	XIV
0.88	KOG3045	YDR083w	viable	IV
0.88	KOG3003	YOR232w	inviable	XV
0.87	KOG4018	YDR152w	viable	IV
0.87	KOG3786	YLR418c	viable	XII
0.87	KOG3789	YEL062w	viable	V
0.86	KOG0809	YOL018c	viable	XV
0.86	KOG4093	YPL225w	viable	XVI
0.86	KOG3015	YJL180c	viable	X
0.86	KOG2487	YPR056w	inviable	XVI
0.85	KOG0438	YEL050c	viable	V
0.85	KOG0645	YDR267c	inviable	IV
0.85	KOG2851	YIR008c	inviable	IX
0.85	KOG2267	YKL045w	inviable	XI
0.84	KOG2732	YJR006w	inviable	X
0.84	KOG2021	YKL205w	viable	XI
0.83	KOG0991	YOL094c	inviable	XV
0.83	KOG3224	YPR040w	viable	XVI
0.83	KOG2994	YML021c	viable	XIII
0.82	KOG3103	YGR172c	inviable	VII
0.82	KOG1598	YGR246c	inviable	VII
0.82	KOG0436	YGR171c	viable	VII
0.81	KOG2326	YMR106C	viable	XIII
0.81	KOG1355	YNL220w	viable	XIV
0.81	KOG1741	YPR166c	viable	XVI
0.80	KOG3381	YHR122w	inviable	VIII
0.79	KOG3244	YDR204w	viable	IV
0.79	KOG1534	YLR243w	inviable	XII
0.78	KOG3229	YKL041w	viable	XI
0.77	KOG3438	YNL113w	inviable	XIV
0.77	KOG1069	YGR095c	inviable	VII
0.76	KOG3364	YIL065c	viable	IX
0.76	KOG0989	YJR068w	inviable	X
0.75	KOG3911	YDR087c	inviable	IV
0.73	KOG3104	YDR005c	viable	IV
0.73	KOG0304	YNR052c	viable	XIV
0.73	KOG3341	YPL002c	viable	XVI
0.72	KOG3059	YPL076w	inviable	XVI
0.71	KOG3259	YJR017c	inviable	X
0.71	KOG3313	YGR078c	viable	VII
0.70	KOG1750	YNR036c	viable	XIV
0.70	KOG0396	YIL097w	viable	IX
0.70	KOG3240	YPR113w	inviable	XVI
0.69	KOG1173	YKL022c	inviable	XI
0.68	KOG2626	YLR015w	viable	XII
0.66	KOG1299	YGL095c	viable	VII
0.65	KOG3327	YJR057w	inviable	X
0.62	KOG1746	YOR103c	inviable	XV
0.61	KOG3159	YJL046w	viable	X
0.56	KOG0325	YLR239c	viable	XII
0.50	KOG3063	YJL053w	viable	X
0.50	KOG0282	YDR364c	viable	IV
0.48	KOG2874	YCL059c	inviable	III
0.44	KOG4017	YMR201c	viable	XIII
0.36	KOG3228	YDR163w	viable	IV
0.35	KOG0551	YBR155w	inviable	II
0.24	KOG0285	YPL151c	inviable	XVI
0.08	KOG2441	YAL032c	inviable	I

**Figure 1:**
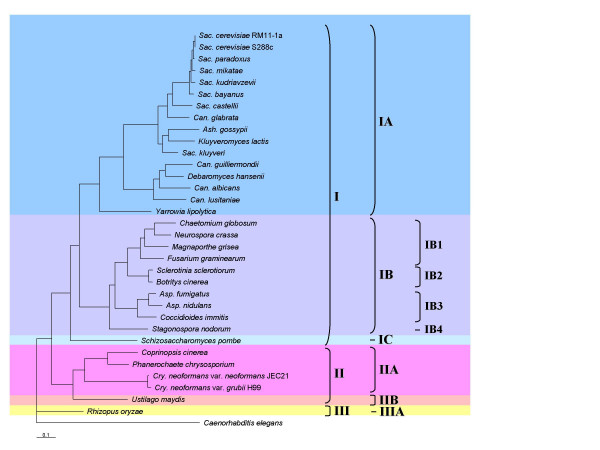
Phylogenetic relationship of 33 complete fungal genomes. The same tree topology is given by concatenation of 30, 40, 50, 60 and 64 KOG proteins with correlation values >0.50 when compared to reference KOG2671 distance matrix. Asp. = Aspergillus, Can. = Candida, Cry. = Cryptococcus, Sac. = Saccharomyces, Ash. = Ashbya. Phyla: I = Ascomycota, II = Basidiomycota, III = Rhyzomycota. Subphyla: IA = Saccharomycotina, IB = Pezizomycotina, IC = Taphrinamycotina, IIA = Agaricomycotina, IIB = Ustilaginomycotina, IIIA = Mucormycotina. IB1 = Sardariomycetes, IB2 = Letiomycetes, IB3 = Eurotiomycetes, IB4 = Dothideomycetes. Support values indicated on the branches were obtained by bootstrap analysis using 100 replicates. * indicates support values of 98–100%.

Comparing the CCC values of a 531 × 531 distance matrices analyzed before [14] using Pearson's correlation, indicated that KOG2671 represents the single copy protein with the highest correlation value of 0.96 (Additional file 2). This KOG2671 protein (putative RNA methylase KOG annotation) corresponds to ORF YOL124c of *S. cerevisiae *[Catalytic subunit of an adoMet-dependent tRNA methyltransferase complex (Trm11p-Trm112p), required for the methylation of the guanosine nucleotide at position 10 (m2G10) in tRNAs; contains a THUMP domain and a methyltransferase domain]. The CCC values of the remaining 69 single copy KOGs were compared with that of KOG2671. Any of the subsequent five single protein KOGs present in the list of 531 KOG proteins [14], namely KOG2728 (Uncharacterized conserved protein with similarity to phosphopantothenoylcysteine synthetase/decarboxylase), KOG0991 (Replication factor C, subunit RFC2), KOG0340, (ATP-dependent RNA helicase), KOG0809 (SNARE protein TLG2/Syntaxin 16), and KOG3786 (RNA polymerase II assessory factor Cdc73p), could be used as a starting point for this comparison, because the correlation values ranged between 0.95 and 0.96 (Additional file 2). The correlation values between the distance matrix of KOG2671 and that of each of the remaining 69 KOG proteins ranged from 0.08 to 0.93 (Table 1), and were statistically significant (Additional file 3). The majority of the KOGs (i.e. 64 from 70 KOGs) gave correlation values higher than 0.50 (Table 1). As an example, we constructed a phylogenetic tree based on concatenation of these 64 KOGs (Fig. 1), which is in accordance with previously published trees. Four KOGs gave CCC values below 0.36 (Table 1), thus indicating that they have different phylogenetic signals. This is sustained by the resulting phylogenetic tree showing a different topology (Additional file 4) if compared with that based on 64 KOGs (Fig. 1). For instance, the Pezizomycotina formed a sister clade to the Basidiomycetes and, *S. pombe *occured as a basal lineage to both of them, but without statistical support (Additional file 4).

**Figure 2:**
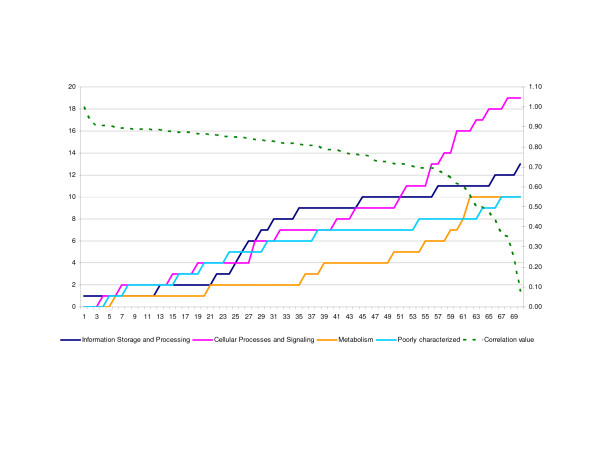
Graph representing the number of concatenated KOGs (x-axis) per functional KOG category (information storage and processing; cellular processes and signaling; metabolism; poorly characterized), and the correlation values between KOG2671 distance matrix and each distance matrix of the 70 KOGs (right y-axis). The left y-axis illustrates the cumulative values of each KOG functional category when they are concatenated. The corresponding KOG protein number in x-axis is listed in the Table 1 and the corresponding functional category is in Supplemental Table 1.

Among the KOG proteins with CCC values above 0.50, are many proteins involved in cellular processes and signaling. The other tree KOG categories [20], namely information storage and processing, metabolism, and poorly characterized categories seem to be less informative (Fig. 2). When the KOG proteins are concatenated in increasing numbers (e.g. the 10 with the highest CCC values; the 20 with the highest CCC values and so on) it can be seen that the CCC values remains above 0.8 until 44 proteins have been concatenated (Fig. 2). Thereafter, the CCC values showed a sharp decline, indicating that the KOG proteins 44–64 have different phylogenetic signals. Interestingly, the topology of the phylogenetic trees stabilizes after the concatenation of 40 proteins (Additional file 5). After concatenation of only 10 and 20 proteins the lineages with *C. glabrata, S. kluyveri, K. lactis *and *A. gossypii*, and that of *C. lusitaniae, D. hansenii, C. guilliermondii *and *C. albicans*, and finally the Euascomycete lineage of *C. globosum, N. crassa, M. grisea *and *F. graminearum *showed varying topologies (Additional file 5). Bootstrap values of most branches were high irrespective the number of proteins concatenated (Fig. 1, Additional file 5). However, for two branches, labeled 7 and 9 in Additional file 5, that received lower bootstrap values, the maximum value (85%) was obtained after concatenation of 40 KOG proteins. The *A. gossypii-K. lactis-Sac. kluyveri *lineage (labeled as branches 4 and 5 in Additional file 5) received only low support, and this was even true after concatenation of 531 orthologues [14]. This most likely indicates that further improvement can only be obtained by further species sampling in this lineage. Summarizing we estimate that 40–45 concatenated single copy protein KOGs are needed to fully resolve fungal TOL. Below this number the tree topology may be different, and above this number the CCC values as well as the support values tend to drop.

### Reevaluating fungal TOL

In all phylogenetic trees using 10–64 concatenated single KOG proteins, the clades I, II and III correspond to the Ascomycota, Basidiomycota and Zygomycota phyla, respectively (Fig. 1, Additional file 5), thus agreeing with analyses using a supertree method [16], a super alignment using restricted orthology [21], and concatenation of six genes [10], and 153 [15] and 531 proteins [14], respectively. Not surprisingly, the Ascomycota formed a sister clade to the Basidiomycota, with the Zygomycota forming a basal lineage.

The Ascomycota are well represented because of the number of available sequenced genomes, and is subdivided into subphyla Pezizomycotina, Saccharomycotina and Taphrinamycotina (Fig. 1). The Saccharomycotina (clade IA) formed a sister group to the Pezizomycotina (clade IB), with Taphrinamycotina (clade IC) forming a basal lineage to both (Fig. 1). The resolution of the Saccharomycotina and Pezizomycotina is in agreement with previous phylogenomic analyses [10,16,21].

The phylogenetic structure of the subphylum Saccharomycotina in our tree (Fig. 1) is similar to that based on a combination of 153 protein families [15], but slightly differs from that based on an analysis using six combined genes [10]. Noticeable differences are the positions of *D. hansenii*, *C. guilliermondii*, *C. lusitaniae *and *C. albicans*. In our analysis and the study of Fitzpatrick et al. [16], these four species formed a single cluster (Fig. 1), while in the six-gene analysis [10], *C. albicans *clusters with *C. guilliermondii*, and *D. hansenii *with *C. lusitaniae*.

Within the Saccharomycotina, seven species evolved after WGD [1], namely *S. cerevisiae*, *S. bayanus*, *S. castellii*,*S. kudriavzevii*, *S. mikatae*, *S. paradoxus *and *C. glabrata*. The basal position of *C. glabrata *among these species agrees with results from Fitzpatrick et al. [16], but only after removal of fast evolving site classes in their dataset. The phylogenetic structure of the *Saccharomyces sensu stricto *species, *S. cerevisiae*, *S. paradoxus*, *S. mikatae*, *S. kudriavzevii *and *S. bayanus *corroborated with previous results of Rokas et al. [12] and Kuramae et al. [14], but was found to be somewhat different if compared with data obtained by comparative genomic hybridization (CGH) [22] and a four-gene analysis [8] (Additional file 6). In the CGH study the positions of *S. mikatae *and *S. kudriavzevii *differ, whereas in the four-gene analysis *S. cerevisiae*, *S. paradoxus *and *S. mikatae *occupied different positions.

The subphylum Pezizomycotina is divided into four clades: Sordariomycetes (clade IB1), Leotiomycetes (clade IB2), Eurotiomycetes (clade IB3) and Dothideomycetes (clade IB4) (Fig. 1). The phylogenetic positions of the Sordariomycetes, Leotiomycetes and Dothiomycetes have been a matter of controversy. According to our analysis, the Sordariomycetes and Leotiomycetes are sister clades, which is in agreement with other studies [10,16,23], although the tree in the latter study was only weakly supported. All these results are, however, in disagreement with data resulting from a four-gene analysis [9], in which the Dothideomycetes occurred as a sister clade to the Sordariomycetes. The position of *Stagonospora nodorum *(Dothiomycetes) as a basal lineage in the Pezizomycetes is highly supported in our analysis (> 90% bootstrap) (Fig. 1, Additional file 5) and corroborates with data from James et al. [10] and Robbertse et al. [17] who used maximum parsimony. However, in analyses based on a supertree method, and 153 concatenated proteins [15] and a four-gene analysis [9], *S. nodorum *was found to be positioned next to the Eurotiomycetes [9,16,21] or closely to the Sordariomycetes and Leotiomycetes [16].

All analyses using concatenated proteins with CCC values above 0.50 (Fig. 1, Additional file 5) positioned *S. pombe *(Taphrinomycotina) as a basal lineage within the phylum Ascomycota, which is in concordance with many other studies [10,14-16,21] using different sets of genes or orthologous proteins and different methods of analysis [15]. However, in another study [15], part of the concatenated orthologues resulted in a different position, which was explained by assuming a different evolutionary origin of these proteins.

The topology of the few basidiomycetous species included, representing only two subphyla Agaricomycotina (clade IIA with *Coprinopsis cinerea*, *Phanerochaete chrysosporium*, *Cryptococcus neoformans *var. *neoformans*, *C. neoformans *var. *grubii*) and Ustilaginomycotina (clade IIB with *Ustilago maydis*) (Fig. 1, Additional file 5) corroborates with previous studies [10,16].

Our method of protein selection using CCC values of individual protein distance matrices seems an useful approach as the resulting phylogenetic trees are largely in agreement with those published elsewhere, and, importantly, most of the branches are well supported. The resulting selection of proteins may also be used to analyze the majority of fungal species for which a full genome is not yet available in order to improve our understanding of fungal TOL.

The performance of our method, if compared to the recent AFTOL study [10], was assessed by comparing CCC values between the protein distance matrix of reference KOG2671 and that based on the combined data set of six AFTOL genes. The correlation value obtained was 0.73, thus indicating that our reference protein has a rather similar phylogenetic signal if compared to the AFTOL genes. However, the inclusion of more genes increases the phylogenetic signal as demonstrated in our analysis (Fig. 1, Additional file 5), which may contribute to the resolution of discordant branches, such as that of *A. gossypii-K. lactis-S. kluyveri *clade.

## Conclusion

In short, the set of proteins resulting from our studies presents a good selection to be elaborated in further studies on fungal TOL, which may include many non-sequenced species. As the proteins were selected across the fungal kingdom and because they represent single KOG proteins, they may also be suitable for the development of molecular barcodes. This proposed method is universal and can be extended easily to bacterial and archaeal TOLs as well as other eukaryote lineages of TOL.

## Methods

### Assignment of genomes to KOG

In this study we used the complete genomes of 33 fungal and one metazoa (*Caenorhabditis elegans*) (Table 2). The group orthology framework presented in the KOG database [20] was the basis of our analyses. KOGs of *Caenorhabditis. elegans*, *Saccharomyces cerevisiae *S288c and *Schizosaccharomyces pombe *were obtained from the KOG database [24]. Thirty one proteomes (*Ashbya gossypii*, *Aspergillus fumigatus, Asp. nidulans, Botritys cinerea*, *Candida albicans*, *Can. glabrata*, *Can. guilliermondii, Can. lusitaniae, Chaetomium globosum, Coccidioides immitis, Coprinopsis cinerea, Cryptococcus neoformans *var. *neoformans*, *Cryp. neoformans *var.*grubii*, *Debaryomyces hansenii*, *Fusarium graminearum*, *Kluyveromyces lactis*, *Magnaporthe grisea*, *Neurospora crassa*, *Phanerochaete chrysosporium, Rhizopus oryzae, Saccharomyces cerevisiae *RM11-1a, *Sac. bayanus*, *Sac. castellii*, *Sac. kluyveri*, *Sac. kudriavzevii*, *Sac. mikatae*, *Sac. paradoxus*, *Sclerotinia sclerotiorum, Stagonospora nodorum, Ustilago maydis *and *Yarrowia lipolytica *were assigned for orthologies using the STRING program as described before [25].

**Table 2:** Genome sources, genome size (Mb), number of KOGs assigned to each genome used in the study

**Genome**	**Strain**	**Genome size (Mb)**	**Number of KOG**	**Location**
*Ashbya gossypii*	ATCC10895	7	2,592	Zoologisches Institut der Univ. Basel, Switzerland
*Aspergillus fumigatus*	Af293	30	3,182	TIGR
*Aspergillus nidulans*	FGSC A4	31	2,982	Broad Institute
*Botritis cinerea*	B05.10	38	3,191	Broad Institute
*Caenorhabditis elegans*		100	4,235	Welcome Trust Sanger Institute
*Candida albicans*	SC5314	16	2,636	Stanford University
*Candida glabrata*	CBS138	13	2,505	Genolevures
*Candida guilliermondii*	ATCC6260	12	2,750	Broad Institute
*Candida lusitaniae*	ATCC42720	16	2,742	Broad Institute
*Chaetomium globosum*	CBS148.51	36	3,144	Broad Institute
*Coccidioides immitis*	RS	28.78	3,137	Broad Institute
*Coprinopsis cinereus*	Okayama 7 (#130).	37.5	3,210	Broad Institute
*Cryptococcus neoformans var. neoformans*	JEC21	24	2,876	TIGR
*Cryptococcus neoformans var. grubii*	H99	20	3,074	Broad Institute
*Debaryomyces hansenii*	CBS767	12.22	2,760	Genolevures
*Fusarium graminearum*	PH-1 (NRRL 31084)	36	3,063	Broad Institute
*Kluyveromyces lactis*	CLIB210	10.69	2,596	Genolevures
*Magnaporthe grisea*	70-15	40	2,917	Broad Institute
*Neurospora crassa*	N-150	40	2,962	Broad Institute
*Phanerochaete chrysosporium*	RP78	30	2,945	DOE Joint Genome Institute
*Rhizopus oryzae*	RA99–880	40	3,310	Broad Institute
*Saccharomyces bayanus*	MCYC623	12	2,560	Stanford University
*Saccharomyces castellii*	NRRL Y-12630	10.2	2,390	Stanford University
*Saccharomyces cerevisiae*	RM11-1a	12	2,665	Broad Institute
*Saccharomyces cerevisiae*	S288c	12.07	2,668	Welcome Trust Sanger Institute
*Saccharomyces kluyveri*	NRRL Y-12651	10.2	1,747	Stanford University
*Saccharomyces kudriavzevii*	IFO1802	10.6	1,855	Stanford University
*Saccharomyces mikatae*	IFO1815	12	2,557	Stanford University
*Saccharomyces paradoxus*	NRRLY-17217	12	2,592	Stanford University
*Saccharomyces cerevisiae*	S288C	13	2,668	Stanford University
*Schizosaccharomyces pombe*	Urs Leupold 972 h^-^	14	2,762	Welcome Trust Sanger Institute
*Sclerotinia sclerotiorum*	1980	38	3,219	Broad Institute
*Stagonospora nodorum*	SN15	37.1	3,324	Broad Institute
*Ustilago maydis*	521	20	2,850	Broad Institute
*Yarrowia lipolytica*	CLIB99	20–21	2,699	Genolevures

### Comparison of KOGs represented by single protein

In order to avoid problems of paralogy we selected only those 70 KOGs represented by a single protein shared by 33 complete fungal genomes. First, each protein from the list of the KOGs that fulfilled this criterion was aligned by Clustal X [26]. Second, poorly aligned positions and divergent regions in each KOG alignment were removed by using Gblocks 0.91b [27]. The threshold parameters used were: minimum number of sequences for a conserved position = 50% of the number of sequences + 1, minimum number of sequences for a flank position = 85% of the number of sequences, maximum number of contiguous nonconserved positions = 8, minimum length of a block = 10, not allowed gap positions, use similarity matrices. Third, the distance matrix (percent divergence) of each KOG protein was calculated between all pairs of sequences from a multiple alignment of each KOG. Finally, each KOG protein distance matrix was compared to each other (70 × 70) by Pearson's correlation.

### Selection of the reference KOG distance matrix

The distance matrices of the 531 KOGs used by Kuramae et al. [14] were calculated. Then, the correlation matrix values between distance matrices were determined by Pearson's correlation as described. To find the KOG distance matrix to be used as reference we selected the single copy KOG protein with the highest correlation value. This reference distance matrix was then compared to the distance matrices of the remaining 69 KOGs selected.

### Phylogenetic analysis

KOG distance matrices with correlation values higher than 0.50 when compared to the reference KOG distance matrix were concatenated, aligned, the poorly aligned regions removed, and a phylogenetic analysis was done by Maximum Likelihood **(**PHYML) [28]. The amino acid model substitution used was JTT [29]. The number of substitution rate categories was 2. The model of rate heterogeneity was Gamma distribution rates with 4 categories. We used *Caenorhabditis elegans *as outgroup for all phylogenetic trees reconstructions. Groups of 10, 20, 30, 40, 50, 60 and 64 KOGs protein according to decreasing cophenetic correlation values were selected, subsequently used to build phylogenetic trees, and their support values assessed using 100 replicates.

### Comparison KOG reference and AFTOL combined genes

For this comparison we used 24 genomes present in AFTOL for which entire genome data are available to calculate the distance matrix of the alignment from AFTOL [30]. The six combined genes distance matrix from AFTOL and the distance matrix of our reference KOG2671 were compared by Pearson's correlation.

## Abbreviations

KOG: Clusters of orthologous groups for eukaryotic complete genomes.

## Authors' contributions

EEK participated in the design of the study, analyses and drafted the manuscript.

VR participated in the design of the study, analyses and drafted the manuscript.

CEE participated in the sequence alignment and drafted the manuscript.

TB participated in the design of the study, analyses and drafted the manuscript.

All authors contributed to the final manuscript preparation.

## Supplementary Material

Additional file 1 Correlation values of KOG distance matrices compared to KOG2671, KOG functional category, the corresponding single protein KOGs to the systematic name, standard name, description, systematic deletion and chromosome number of ORFs of *Saccharomyces cerevisiae *according to [19]. Click here for file

Additional file 2 Correlation values of the comparison between KOGs distance matrices from a set of 531 proteins used in the study of Kuramae et al., 2006 [14]. Click here for file

Additional file 3 Correlation values obtained by Pearson's correlation by comparing the distance matrix of KOG2671 with each distance matrix of 69 KOGs, non-directional probability and T- distribution. Click here for file

Additional file 4 Phylogenetic tree based on concatenation of four KOG proteins with correlation below 0.36. The four KOGs distances matrices were compared with KOG2671 reference distance matrix. Click here for file

Additional file 5 Phylogenetic trees based on concatenation of 20, 30, 40, 50, 60 and 64 KOG proteins with correlation values higher than 0.50. Branches with different topologies obtained after concatenation of 10 or 20 proteins are indicated separately. Support values indicated on the branches were obtained by bootstrap analysis using 100 replicates. * indicates support values of 98–100%. In the Table support values obtained after 10, 20, 30, 40, 50, 60 and 64 proteins (from left to right) are indicated for those branches (labeled 1–12 in tree and table) that received support < 98% in at least one of the sets analyzed are indicated. Note the low to moderate support for lineages 4, 5, 7 and 9. Overall the bootstrap values tend to increase until concatenation of 40 to 50 KOG proteins, but this is also lineage dependent (compare e.g. lineages 7, 9 and 12). Click here for file

Additional file 6 Topological differences in phylogenetic trees of the *Saccharomyces sensu stricto *lineage as inferred from various publications. Click here for file
