# Supplementary material for: Cophenetic correlation analysis as a strategy to select phylogenetically informative proteins: an example from the fungal kingdom
Source: BMC Evol Biol. 2007 Aug 9;7:134. doi: 10.1186/1471-2148-7-134 (PMC2045111; doi:10.1186/1471-2148-7-134)
Supplement: Additional file 4 — Phylogenetic tree based on concatenation of four KOG proteins with correlation below 0.36. The four KOGs distances matrices were compared with KOG2671 reference distance matrix. [file 1471-2148-7-134-S4.ppt]

## Slide 1
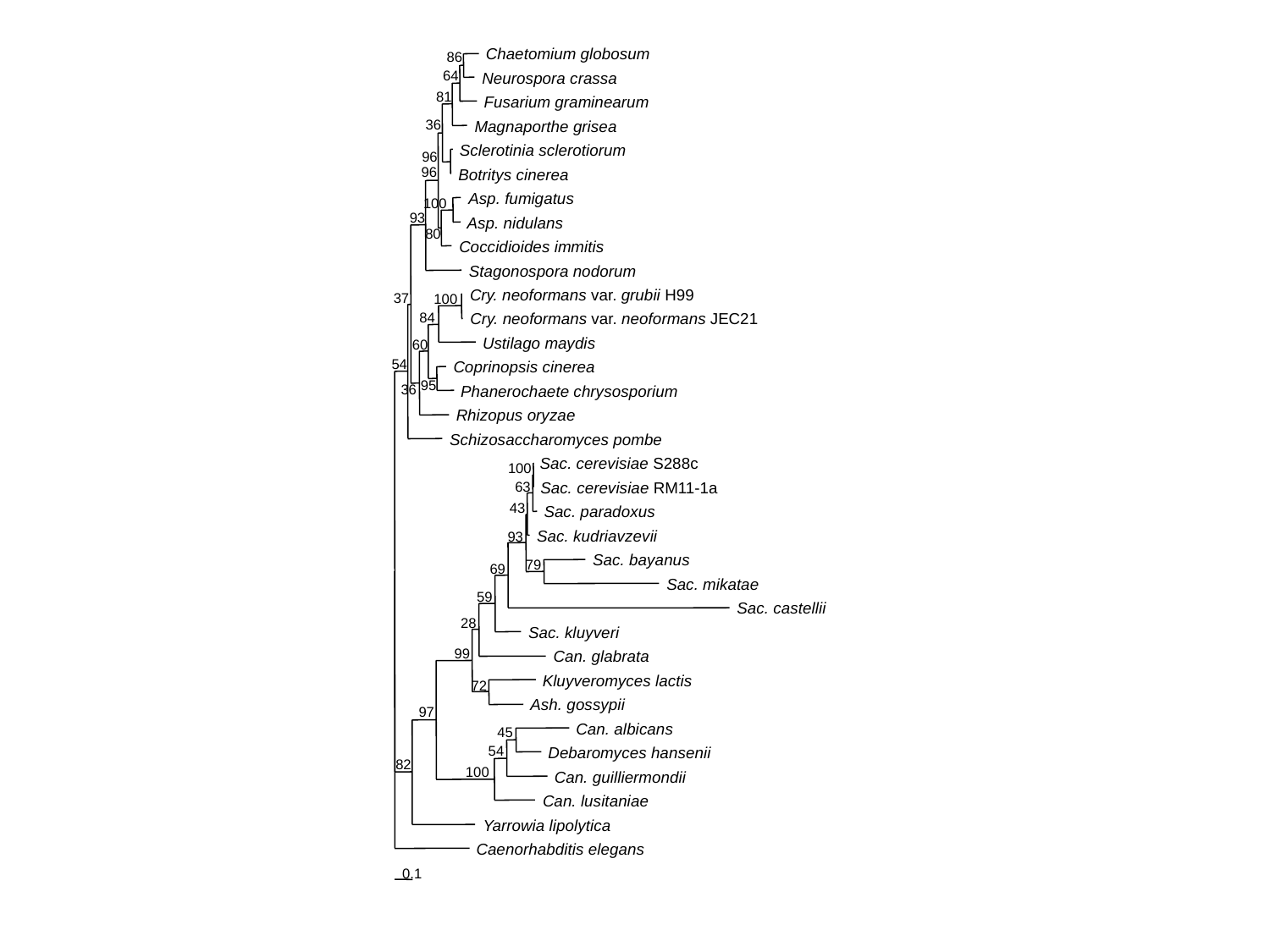

Chaetomium globosum
86
64
Neurospora crassa
81
Fusarium graminearum
36
Magnaporthe grisea
Sclerotinia sclerotiorum
96
96
Botritys cinerea
Asp. fumigatus
100
93
Asp. nidulans
80
Coccidioides immitis
Stagonospora nodorum
Cry. neoformans var. grubii H99
37
100
84
Cry. neoformans var. neoformans JEC21
Ustilago maydis
60
54
Coprinopsis cinerea
95
36
Phanerochaete chrysosporium
Rhizopus oryzae
Schizosaccharomyces pombe
Sac. cerevisiae S288c
100
63
Sac. cerevisiae RM11-1a
43
Sac. paradoxus
Sac. kudriavzevii
93
Sac. bayanus
79
69
Sac. mikatae
59
Sac. castellii
28
Sac. kluyveri
99
Can. glabrata
Kluyveromyces lactis
72
Ash. gossypii
97
Can. albicans
45
54
Debaromyces hansenii
82
100
Can. guilliermondii
Can. lusitaniae
Yarrowia lipolytica
Caenorhabditis elegans
0.1
