# Supplementary figures and images for: Cophenetic correlation analysis as a strategy to select phylogenetically informative proteins: an example from the fungal kingdom
Source: BMC Evol Biol. 2007 Aug 9;7:134. doi: 10.1186/1471-2148-7-134 (PMC2045111; doi:10.1186/1471-2148-7-134)

30-64 KOGs

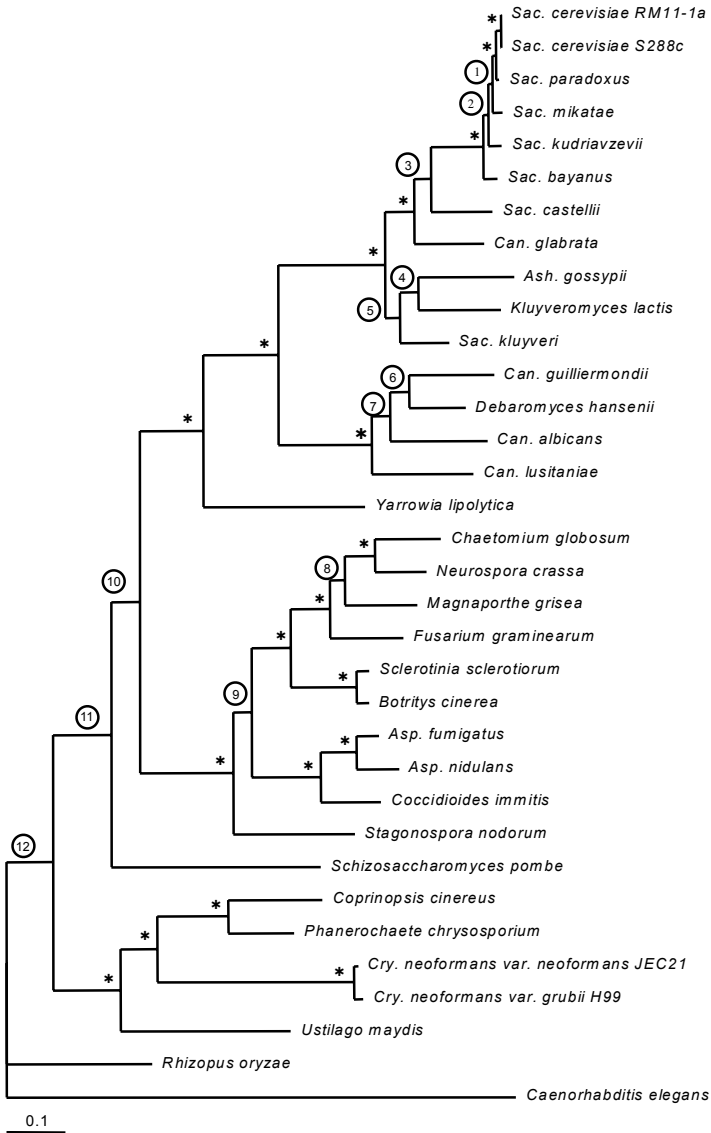

10 KOGs

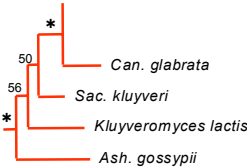

20 KOGs

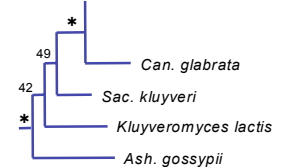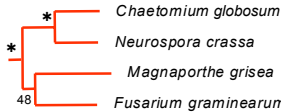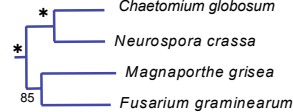

| Concatenated KOGs |    |    |    |    |    |    |    |
|-------------------|----|----|----|----|----|----|----|
|                   | 10 | 20 | 30 | 40 | 50 | 60 | 64 |
| 1                 | 58 | 93 | 90 | *  | *  | *  | *  |
| 2                 | 77 | 75 | 87 | *  | *  | *  | *  |
| 3                 | 69 | 84 | 58 | 76 | 88 | 81 | 80 |
| 4                 | -  | -  | 46 | 54 | 67 | 72 | 62 |
| 5                 | -  | -  | 44 | 40 | 59 | 48 | 36 |
| 6                 | 77 | -  | 72 | *  | 97 | *  | *  |
| 7                 | 49 | -  | 63 | 85 | 79 | 63 | 69 |
| 8                 | -  | -  | -  | 76 | 81 | 72 | 84 |
| 9                 | 54 | 89 | 77 | 84 | 73 | 55 | 59 |
| 10                | 58 | 82 | 82 | 80 | *  | *  | *  |
| 11                | 79 | *  | 93 | *  | *  | *  | *  |
| 12                | -  | 60 | 86 | 86 | 84 | 91 | 86 |

Supplement: Additional file 5 — Phylogenetic trees based on concatenation of 20, 30, 40, 50, 60 and 64 KOG proteins with correlation values higher than 0.50. Branches with different topologies obtained after concatenation of 10 or 20 proteins are indicated separately. Support values indicated on the branches were obtained by bootstrap analysis using 100 replicates. * indicates support values of 98–100%. In the Table support values obtained after 10, 20, 30, 40, 50, 60 and 64 proteins (from left to right) are indicated for those branches (labeled 1–12 in tree and table) that received support < 98% in at least one of the sets analyzed are indicated. Note the low to moderate support for lineages 4, 5, 7 and 9. Overall the bootstrap values tend to increase until concatenation of 40 to 50 KOG proteins, but this is also lineage dependent (compare e.g. lineages 7, 9 and 12). [file 1471-2148-7-134-S5.pdf]
