# Supplementary material for: Cophenetic correlation analysis as a strategy to select phylogenetically informative proteins: an example from the fungal kingdom
Source: BMC Evol Biol. 2007 Aug 9;7:134. doi: 10.1186/1471-2148-7-134 (PMC2045111; doi:10.1186/1471-2148-7-134)
Supplement: Additional file 6 — Topological differences in phylogenetic trees of the Saccharomyces sensu stricto lineage as inferred from various publications. [file 1471-2148-7-134-S6.pdf]

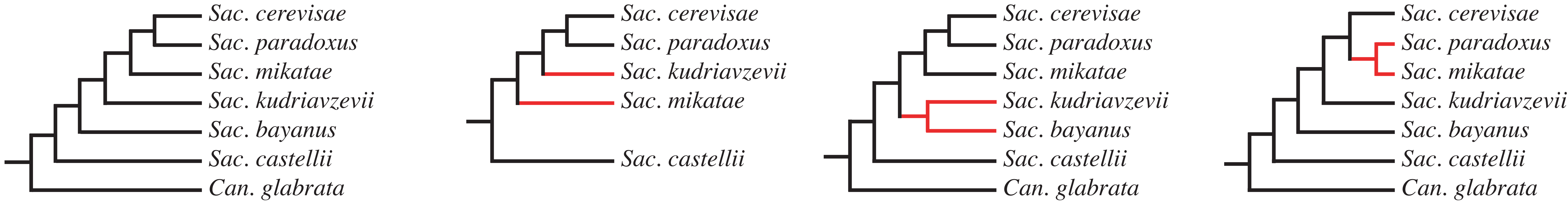

Supported by [14, 15, 16] (Matrix representation with Parsimony and Average Consensus Tree), 18, and this work]

Supported by Comparative Genome Hybridization [21]

Supported by Average Consensus method [16]

Supported by 4-gene phylogeny [8]

Topology changes
